# The known burden of Huntington disease in the North of Scotland: prevalence of manifest and identified pre-symptomatic gene expansion carriers in the molecular era

**DOI:** 10.1007/s00415-021-10505-w

**Published:** 2021-04-15

**Authors:** Georgios Kounidas, Heather Cruickshank, Stavroula Kastora, Stella Sihlabela, Zosia Miedzybrodzka

**Affiliations:** 1 Medical Genetics Group, School of Medicine, Medical Sciences, Nutrition and Dentistry, University of Aberdeen, Aberdeen, AB25 2ZD UK; 2 North of Scotland Regional Genetics Service, Aberdeen Royal Infirmary, Clinical Genetics Centre, Foresterhill, Aberdeen, AB25 2ZA UK

**Keywords:** Huntington Disease, Prevalence, Scotland, General practice

## Abstract

**Background:**

Huntington disease prevalence was first estimated in Grampian, northern Scotland in 1984. Molecular testing has since increased ascertainment.

**Objective:**

To estimate the prevalence of manifest Huntington disease and identified pre-symptomatic gene expansion carriers (IPGEC) in northern Scotland, and estimate the magnitude of biases in prevalence studies that rely upon routine coding in primary care records.

**Methods:**

Cases were ascertained using North of Scotland genetic laboratory, clinic, and hospital records. Prevalence was calculated for manifest and IPGEC on 01/07/2016 and 01/01/2020 and compared with local published data.

**Results:**

The prevalence of manifest Huntington disease in northern Scotland in 2020 was 14.6 (95% CI 14.3–15.3) per 100,000, and of IPGEC was 8.3 (95% CI 7.8–9.2) per 100,000. Whilst the population of northern Scotland decreased by 0.05% between 2016 and 2020, the number of manifest and identified pre-symptomatic gene expansion carriers increased by 7.4% and 23.3%, respectively. Manifest disease in Grampian increased by 45.9% between 1984 and 2020. More women than men had a diagnosis. General Practice coding underestimated symptomatic molecularly confirmed prevalence by 2.2 per 100,000 people.

**Conclusion:**

Even in an area with previously high ascertainment, there has been a 45.9% increase in manifest Huntington disease over the last 30 years. Within our catchment area, prevalence varies between health board regions with similar community-based services. Such variation in prevalence could have major drug cost and service delivery implications, especially if expensive, complexly administered therapies prove successful. Health services should gather accurate population-based data on a regional basis to inform service planning.

## Introduction

Huntington disease (HD) is an autosomal dominant adult-onset neurodegenerative disorder caused by the expansion of a glutamine (CAG) repeat tract within exon 1 of the Huntington (*HTT*) gene [1]. Although to date, there is no disease-modifying treatment, promising results from a trial of anti-sense oligonucleotide (ASO) therapies are offering affected families hope [2, 3]. Thus, having an accurate prevalence estimate for the population is increasingly important for service planning.

The North of Scotland comprises a relatively large geographic area of the UK, with a rurally distributed population. Health services free at the point of delivery are administered through two major health board areas (NHS Grampian and NHS Highland) and three remote island health boards (NHS Shetland, NHS Orkney, and NHS Western Isles). Population-based studies of prevalence of genetic disorders in the North of Scotland are facilitated by a detailed family-based electronic record system, integration of the genetic laboratory and clinical services, the Scottish electronic patient record system, and long-standing, accurate, and accessible registration of births, marriages, and deaths.

HD was first reported in northern Scotland in the Black Isle region (Highland health board) in 1930 [4]. Careful family-based ascertainment by Simpson and Johnston [4] led to the first formal publication of the prevalence of HD in Grampian in 1989. The health board boundary of Grampian has not changed since the study by Simpson and Johnston [4]. However, in this study, we report data from a much larger geographic area as our clinic now also serves NHS Highland and the other island health boards. In 1989, genetic testing was conducted using linkage analysis, and diagnosis was only confirmed in the presence of characteristic movement disorder with a family history of such. This contrasts with the current direct mutation testing method that counts the number of CAG repeats in the *HTT* gene. For many years, this prevalence of 9.94 per 100,000 people remained amongst the highest recorded, beyond the small population of the lake Maracaibo area in Venezuela [5]. Morrison et al. [6] also reported a similarly high prevalence of 10.6 per 100,000 in Northern Ireland. Since the identification of the *HTT* gene in 1993, awareness of HD has increased [7].

Several studies have used general practice (GP) diagnostic codes to estimate HD incidence and prevalence in research active practices across the UK [8–10]. However, there was no molecular confirmation of the study populations, and observation in our clinic led us to suspect that identified pre-symptomatic gene expansion carriers (IPGEC) might be miscoded as symptomatic, falsely elevating manifest HD prevalence.

The aim of this study was to update the regional prevalence estimates for the purposes of service planning, using routine data, and to calculate a correction factor for improving prevalence estimates based on GP coding.

## Materials and methods

### Patient inclusion criteria

Any individual who was living in the North of Scotland on 01/07/2016 and on 01/01/2020 with 36 or more CAG repeats in *HTT* mutation was included. Such individuals were considered to manifest disease in the presence of characteristic movement disorder assessed by an experienced European Network of Huntington Disease (EHDN) certified rater.

### Sources of ascertainment

Patients were ascertained using NHS Grampian genetic department laboratory and clinic records. Prevalence was estimated for manifest and identified pre-symptomatic gene expansion carriers on 01/07/2016 and on 01/01/2020. Population figures were obtained from published local council demographic data for 2016 and 2019 [11]. NHS patient records were examined to identify the number of CAG repeats on the affected allele for manifest and pre-symptomatic HD, as well as their age and gender. Molecular testing took place in a single ISO accredited genetics laboratory with a special interest in HD. There is only one HD clinic in the North of Scotland; all HD patients ascertained by the regional laboratory and the local family organisation employed community-based nurses, who working closely with the clinic are referred. The clinical lead of the HD service also leads the genetics laboratory, and this fosters joint working and thus supports ascertainment. A very small number of patients resident in the region may be unknown to the clinic if they were tested in another laboratory south of Grampian but in our experience this is very uncommon. There may also be some affected people in the population who have not come forward for diagnosis.

### General practitioner (GP) coding

To assess the accuracy of GP coding, the history section of GP referral letters within the hospital electronic patient record was examined to estimate: (1) the proportion of IPGEC whose GP records were coded “Huntington Disease”; and (2) the proportion of symptomatic HD patients whose GP records were coded "Huntington Disease". A retrospective case note review of North of Scotland genetic clinic records and Trakcare, the NHS Grampian electronic healthcare record was performed. GP coding was examined by retrospective review of GP referral letters held in the available electronic secondary care electronic patient records for manifest HD patients and Identified IPGEC.

### Statistical analysis

Prevalence was calculated by the following equation: [Prevalence (per 100,000 people) = Number of individuals/Population × 100000], for manifest HD and IPGEC, for each NHS health board and for the North of Scotland in total, together with along with binomial 95% confidence intervals (CI) based on Poisson distribution assumption, where Poisson parameter (λ) was defined as λ = k/n. Total events (*k*) were imputed as the total number of patients identified either as manifesting or pre-symptomatic to the population (*n*) of each health board at the examined time.

A paired *t *test was used to compare the rate of manifest and IPGEC individuals between health board areas between 2016 and 2020. The false discovery rate was assessed via a two-stage linear step-up procedure of Benjamini et al. [12]. Bar charts were employed to visualise the prevalence amongst the health boards between 2016 and 2020. Simple linear regression and non-linear fit (Gaussian) tests were employed to compare the pattern and correlation of distributions between NHS Grampian and Highland HD. Significance was assessed by paired two-tailed *t* test analysis. Two-tailed *p* values were calculated and visualised according to the *NJEM* scale, with *p* values < 0.05 being considered as the threshold for statistical significance. Historic prevalence data for NHS Grampian were obtained from published work; the health board boundaries have not changed. Percentage change was calculated according to the following formula: Percentage Change = (ΔN/|N1)|× 100, where Δ*V* = Patient Numbers _2016_-N_2020_.

## Results

### Prevalence estimates

**Fig. 1:**
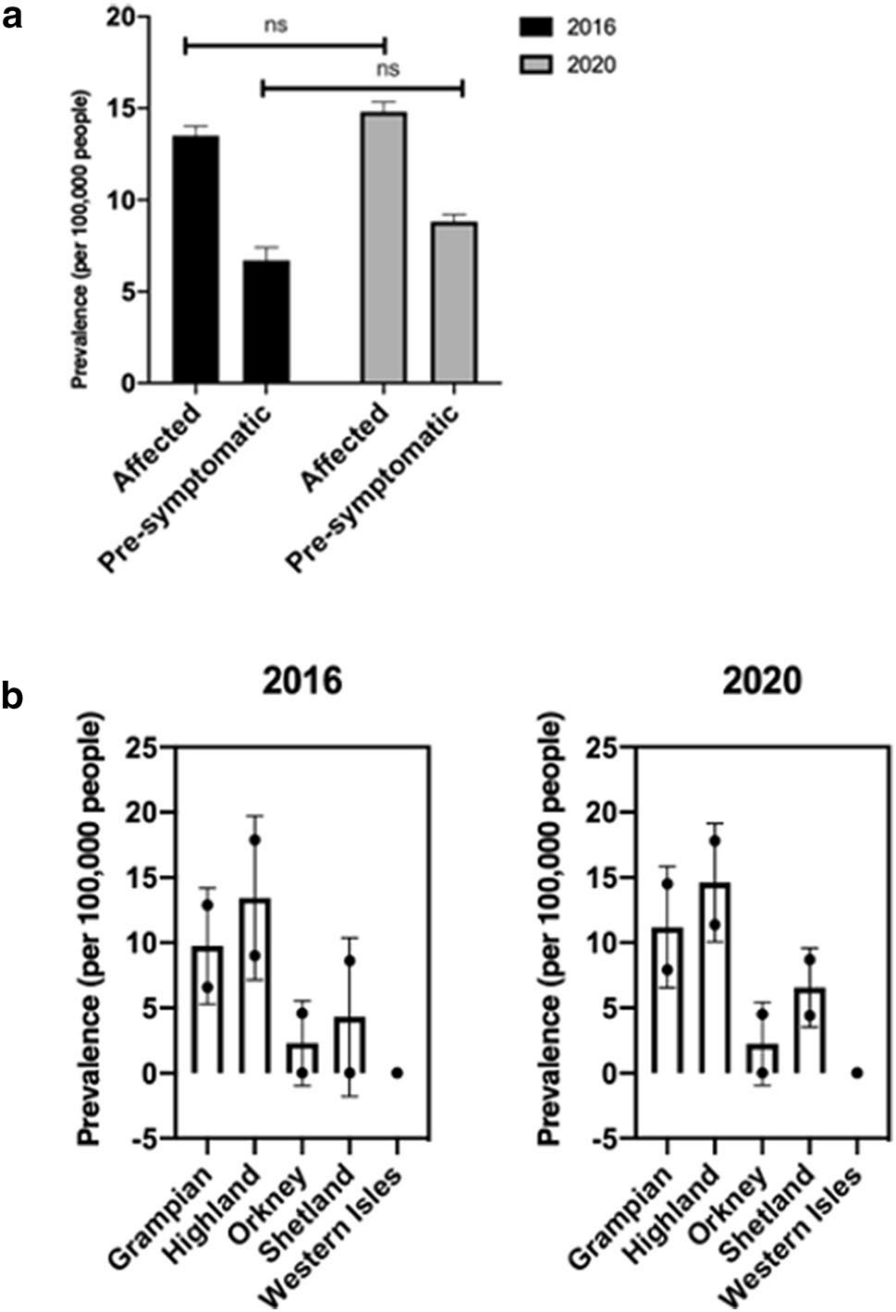
**a** Prevalence of manifest and IPGEC HD in the North of Scotland **b** Bar charts, depicting the HD prevalence amongst health boards between 2016 and 2020

The molecularly confirmed prevalence of manifest HD in northern Scotland in 2020 was 14.6 (95% CI 14.3–15.3) per 100,000, and of IPGEC was 8.3 (95% CI 7.8–9.2) per 100,000. Table 1 shows prevalence by NHS health board. In line with Scottish data governance policy, where number of cases was less than 5 the exact data point was not given to protect anonymity. The IPGEC prevalence data represent the prevalence of individuals who had genetic testing for HD in the population and who were pre-symptomatic at the time of assessment. Highland health board had the highest regional prevalence of molecularly confirmed symptomatic HD and IPGEC (Table 1), but the difference in rate between Grampian and Highland was not statistically significant (p = 0.6) (Fig. 1).

**Table 1:** HD prevalence data for regions in the North of Scotland on 01/07/2016 and on 01/01/2020

Location	HD status	Number of individuals (2020)	Number of individuals (2016)	Population (2020)	Population (2016)	Prevalence (per 100,000 people) (2020) (95% CI)	Prevalence (per 100,000 people) (2016) (95% CI)
Grampian	Manifest	85	76	585,700	587,820	14.5 (13.9–15.1)	12.9 (12.2–13.6)
	Identified Pre-symptomatic Gene Expansion Carrier	46	39			7.9 (7.0–8.8)	6.6 (5.7–7.5)
Highland (excluding Argyll and Bute)	Manifest	42	42	235,830	234,110	17.8 (16.9–18.7)	17.9 (17.0–18.8)
	Identified Pre-symptomatic Gene Expansion Carrier	27	21			11.4 (10.3–12.5)	9 (7.7–10.3)
Island Boards (Orkney, Shetland, Western Isles)	Manifest	< 5	< 5	71,910	71,940	4.17 (3.0–5.0)	4.17 (3.0–5.0)
						1.39 (0.7–2.7)	–
	Identified Pre-symptomatic Gene Expansion Carrier	< 5	< 5				
North of Scotland (Total)	Manifest	< 134	< 123	893,440	893,870	14.6 (14.3–15.3)	13.5 (13.0–14.0)
	Identified Pre-symptomatic Gene Expansion Carrier	< 78	< 65			8.3 (7.8–9.2)	6.7 (6.0–7.4)

Whilst the population in the North of Scotland decreased by 0.05% between 2016 and 2020, the number of manifest HD patients increased by 7.4%, and the number of IPGEC increased by 23.3%. Between 2016 and 2020, three patients moved out of the study area, and 27 known gene positive carriers became manifest. All individuals who had a predictive test and a bad news result remained domiciled in the region on the 2020 prevalence date.

In Grampian, the number of manifest HD cases increased by 11.8%, and the number of IPGEC increased by 17.9%, whilst the population in Grampian decreased by 0.4% between 2016 and 2020. In contrast within Highland, the population increased by 0.7% between 2016 and 2020, and the number of pre-symptomatic patients increased by 28.6% but there was no increase in the number of manifest patients.

The number of cases in each of the Island health boards of Shetland, Orkney and Western Isles was low, and in line with NHS Scotland policy, these data are summarised as “< 5” for these boards combined.

### Clinical and demographic features

#### CAG repeats

**Fig. 2:**
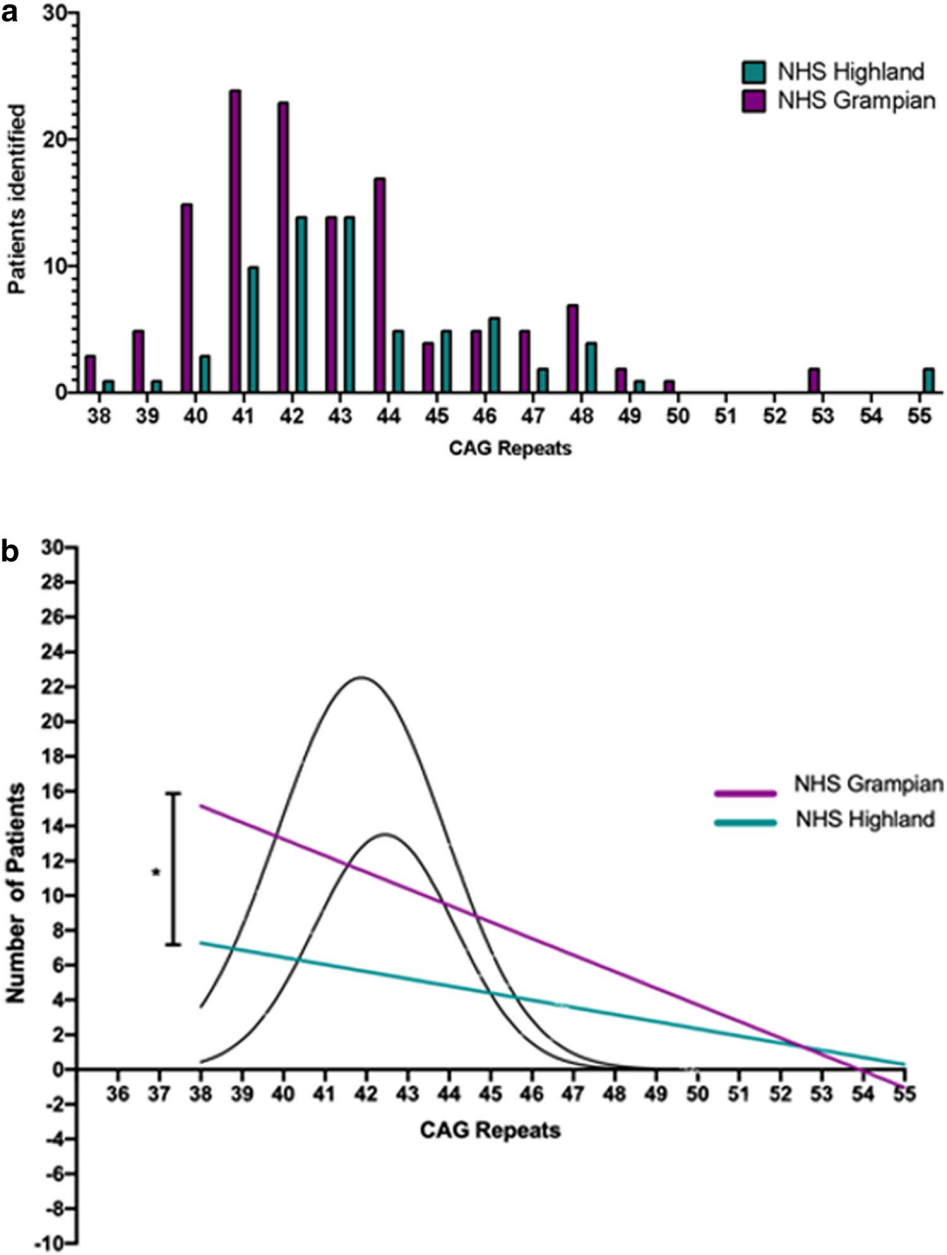
**a** Comparison of CAG repeats in Highland and Grampian populations (manifest and IPGEC HD) on 01/01/2020 **b** Pattern of distribution of CAG numbers between NHS Grampian and Highland HD, paired *t *test p value: 0.0125 (*)

The range of CAG repeat lengths in the affected allele was examined in Grampian and Highland. The percentage of HD population (manifest and IPGEC) from each region for each CAG repeat length was calculated (Fig. 2a). The Pearson correlation test depicted a positively and statistically significant correlation between CAG Grampian and CAG Highland datasets (Pearson *r* = 0.8163, p (two-tailed) = < 0.0001). Larger CAG repeat tracts were more common in Highland compared to Grampian (Grampian median: 42; Highland median: 43) (Fig. 2a), whilst the linear regression analysis amongst the two populations demonstrated that this difference was significant (p value: 0.0125) (Fig. 2b).

### Age and gender distribution

**Fig. 3:**
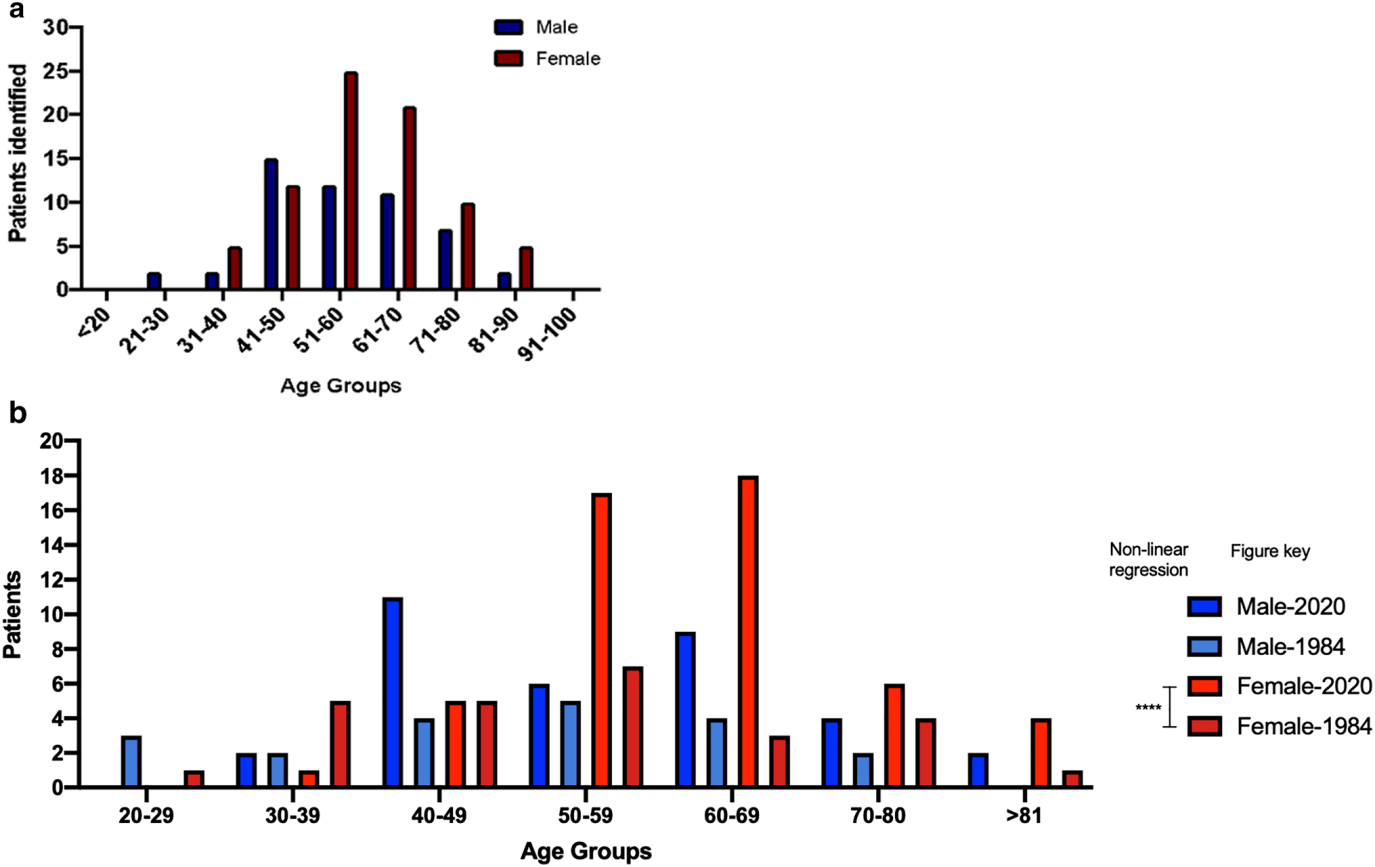
**a** Gender distribution by age of manifest HD on 01/01/2020 in the North of Scotland. **b** Gender distribution by age of manifest HD on 01/01/2020 and on 01/06/1984 in Grampian

Age and gender distribution of manifest HD in the North of Scotland is shown in Fig. 3a. More women than men with manifest HD were ascertained (60.5% vs. 39.5%) on 01/01/2020. Most female individuals belonged to the 51–60 age group, whereas most male individuals belonged to the 41–50 age group. Although no statistically significant difference was found in the age distributions between our study and that of Simpson and Johnston [4], the female-manifest HD has significantly increased since 1984 (*p* = 0.0003) (Fig. 3b).

### General practice (GP) coding

**Fig. 4:**
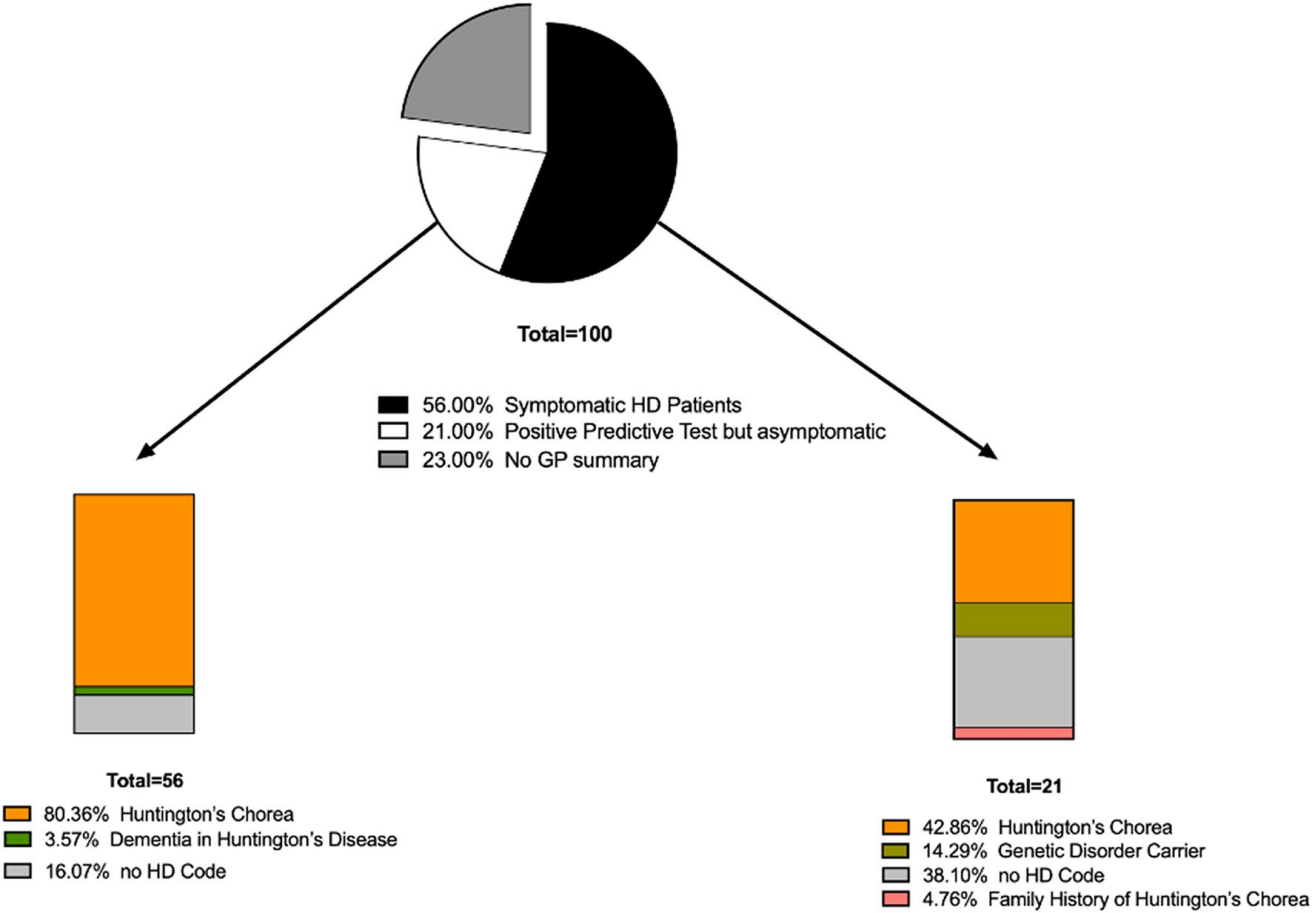
GP Coding of symptomatic and IPGEC HD in NHS Grampian

In GP referral letters for 56 symptomatic HD patients, 80.4% included the code “Huntington’s Chorea”, 3.6% as “Dementia in Huntington’s disease”, and 16% had no HD code. Of the GP referral letters for 21 IPGEC, only 14.3% were correctly coded as "Genetic Disorder Carrier”, whilst 4.7% were coded as “Family History of Huntington’s Chorea.” Moreover, 42.9% were incorrectly coded as affected with “Huntington’s Chorea”, and 38.1% had no HD code at all (Fig. 4).

## Discussion

Here, we describe the rise in the estimates of the prevalence of manifest and IPGEC HD in the North of Scotland in over three decades, based upon data for a whole population. The strengths of this study were that it was population-based, with direct molecular confirmation and case note review in each case, ensuring that only truly symptomatic molecularly confirmed cases were included. Access to genetic laboratory records and referral processes, with a single HD clinic for the region, and embedded links with the family association community-based nurse mean that ascertainment was maximised, and the opportunity to compare with a similarly conducted historic series adds value. An inevitable limitation of such study is that patients tested out with the region who do not attend the local clinic would not be included.

The molecularly verified prevalence of motor-manifest HD in the North of Scotland in 2020 was 14.6 (95% CI 14.3–15.3) per 100,000 manifest individuals, and 8.3 (95% CI 7.8–9.2) per 100,000 IPGEC were known to services. More women were identified with manifest HD than men, particularly in the 51–60 age group. Our prevalence rate is similar to the rate of 13.7 per 100,000 people in Caucasians, reported in British Columbia using similar methods [13].

The prevalence we have observed in the North of Scotland is almost three times greater than reported elsewhere in Europe, North America, and Australia (5.70 per 100,000 people), and more than five times the estimated worldwide prevalence of 2.71 per 100,000 people [14–16]. The widely quoted UK General Practice Research Database (UKGPRD) study estimated UK HD prevalence by examining diagnostic coding in routine clinical records of patients aged 20 and above from 625 of 9800 UK general practices. That study reported a prevalence of 12.3 per 100,000 people over 20 for the UK overall, and 16.1 per 100,000 estimated for Scotland, and only examined 186,902 records in Scotland, compared with the entire North of Scotland population of 870,520 people in this study. In the Scottish census of 2011, 11,184,879 of the population of 5,295,403 were aged 20 and under [11]. Thus, a prevalence of 16.1 per 100,000 in those over 20, only represents an all-age HD prevalence of the order of 12.4 per 100,000 for Scotland, leaving the North of Scotland prevalence of 14.6 per 100,000 as amongst the highest in the UK.

Whilst the population in the North of Scotland decreased by 0.05% between 2016 and 2020, the number of manifest patients increased by 7.4%, and the number of IPGEC increased by 23.3%. It is unlikely that the increase in reported prevalence is due a true increase, it is most likely due to a combination of better ascertainment, and increased longevity in the general population leading to more HD gene expansion carriers, surviving long enough to manifest disease. It should be worth noting that in the interim between the two prevalence dates, there have been significant advances in the pursuit of disease-modifying trials in HD that may have had an impact in the number of people coming forward for testing and/or treatment to have access to these trials [17]. In our practice, we see more individuals seeking earlier diagnosis to benefit from medical and state-funded financial support and care. Moreover, availability of direct mutation testing to identify the disorder in individuals with no known family history of HD, and increased awareness of the condition in the medical profession and amongst the general public may also be contributing factors. The gender effects are likely to be in part be explained by differences in life expectancy. All of the cases of manifest HD reported between the two prevalence dates were known to the clinic as IPGEC. Hence, at least part of the explanation for the increase in the prevalence could be that we diagnosis is now made earlier rather than those with HD living longer.

CAG repeat tracts were longer in cases from Highland than those from Grampian. Historically, these regions had discrete population origins, with Celts from Ireland migrating to replace the Pictish people across Highland but not the east of Grampian some 2000 years ago, and subsequent Viking influence in coastal regions, especially the Northern and Western Isles. More recently, following the early eighteenth century Jacobite wars of independence, major population movements were associated with clearances of land for sheep farming. Forced population movement to marginal coastal lands established the workforce for a herring fishing industry around the Black Isle and the Grampian coastline, with little inward migration until recently. In 1962, Lyon described a cluster of Grampian HD cases, whose origins trace back to the same Highland village [18]. Simpson [19] noted that there may be a founder effect within Grampian and proposed that the condition spread from the Black Isle to other coastal communities with the fishing industry. However, the decline in the fishing industry, the increase in young people seeking higher education, and the new opportunities for work with the oil industry and in the urban conurbations of Inverness and Aberdeen, appear to have mitigated this historic effect, and the cluster is no longer so prominent [20]. We propose that expansion of the CAG repeats in generations derived from the original Black Isle cluster CAG repeat tract has contributed to the pattern of larger repeats being seen in Highland.

It is likely that genetic background also contributes to the high HD prevalence. Kay et al. [21] reported that populations with high frequencies of intermediate alleles (27–35 CAG repeats)- such as Grampian at 3.7%- had high new HD mutation rates. There are three major haplogroups at the HD locus: A, B, and C, with A1 and A2 being the highest risk haplotypes. Warby et al. [22] identified that around 80% of the UK HD population examined had A1 and A2 haplotypes, and that haplotype frequency varied in different populations around the world. Kay et al. [21] reported that, in North Scotland, 64% of HD alleles are A1, 9% A2a, and 18% A2b. The authors hypothesised that the proportion of the A1 allele in a population correlates with the new HD mutation frequency, and this may explain the high prevalence in Northern Scotland, and the UK.

In an autosomal dominant disorder, an equal number of men and women with manifest disease are expected. We, like Simpson and Johnston [4], found manifest females over-represented in the HD population (60.5%). As in most populations, female life expectancy exceeds that of males in the North of Scotland [11]. Although this sex difference in longevity contributes to the observed sex difference in manifest HD, the difference is too great for this to be the sole explanation of this female bias. It is generally thought that women are more likely to seek help and receive healthcare than men [23], but Simpson [19] noted that this is unlikely to be the full explanation, as there was no significant difference between male and female hospital admissions in the region.

Our data suggest that GP diagnostic codes under-represent symptomatic HD patients by 16%, but this is in part compensated for by an over-representation of IPGEC in the symptomatic cohort. Indeed, 43% of IPGEC that were coded as if their disease was manifest added 2.88 per 100,000 people to the prevalence. In the symptomatic group, 16% were not coded as if they were manifest, and this would have underestimated the prevalence by 2.29 per 100,000 people. Overall, our data suggest that the estimates in the data linkage paper [7] underestimated prevalence by around 2.2 per 100,000 people. We propose that other regions could use this correction factor to inform service planning.

The demand for predictive testing may rise further if there is disease-modifying treatment becomes available or even before disease onset. Indeed, we have noticed an increase of predictive testing in the north of Scotland, as over half of the symptomatic HD have had a predictive test.

## Conclusion

The prevalence of symptomatic HD in the North of Scotland has increased over the last 36 years from 9.94 per 100,000 to 14.6 per 100,000 in total, 23.3 per 100,000 people are known to have a pathogenic HD mutation. This prevalence remains amongst the highest recorded. Prevalence can vary between adjoining health board areas, which could have major drug cost and service delivery implications, if expensive, complexly administered, and effective therapies become available. Nevertheless, the rise in reported prevalence in Northern Scotland is much less than recorded elsewhere, suggesting that variation in ascertainment may account for much more of the reported differences in prevalence worldwide than currently assumed. The marked recent rise in ascertainment of identified pre-symptomatic gene expansion carriers reflects interest in treatment research and it is likely these numbers would increase much further, should an effective pre-symptomatic treatment become available.
